# Investigation of the Wind-Induced Airflow Pattern Near the Thies LPM Precipitation Gauge

**DOI:** 10.3390/s21144880

**Published:** 2021-07-17

**Authors:** Enrico Chinchella, Arianna Cauteruccio, Mattia Stagnaro, Luca G. Lanza

**Affiliations:** 1 Department of Civil, Chemical and Environmental Engineering, University of Genova, 16145 Genova, Italy; enrico.chinchella@edu.unige.it (E.C.); arianna.cauteruccio@edu.unige.it (A.C.); mattia.stagnaro@unige.it (M.S.); 2 WMO/CIMO Lead Centre “B. Castelli” on Precipitation Intensity, 16145 Genova, Italy

**Keywords:** precipitation, measurement, non-catching gauges, wind induced bias, computational fluid dynamics, wind tunnel, disdrometer

## Abstract

The airflow velocity pattern generated by a widely used non-catching precipitation gauge (the Thies laser precipitation monitor or LPM) when immersed in a wind field is investigated using computational fluid dynamics (CFD). The simulation numerically solves the unsteady Reynolds-averaged Navier–Stokes (URANS) equations and the setup is validated against dedicated wind tunnel measurements. The adopted k-ω shear stress transport (SST) turbulence model closely reproduces the flow pattern generated by the complex, non-axisymmetric outer geometry of the instrument. The airflow pattern near the measuring area varies with the wind direction, the most intense recirculating flow and turbulence being observed when the wind blows from the back of the instrument. Quantitative parameters are used to discuss the magnitude of the airflow perturbations with respect to the ideal configuration where the instrument is transparent to the wind. The generated airflow pattern is expected to induce some bias in operational measurements, especially in strong wind conditions. The proposed numerical simulation framework provides a basis to develop correction curves for the wind-induced bias of non-catching gauges, as a function of the undisturbed wind speed and direction.

## 1. Introduction

Precipitation is among the most challenging environmental measurements. A wide variety of sensors are used to collect precipitation data and non-catching gauges (hereinafter NCGs) are being developed and increasingly adopted by national weather services (NWS). NCGs differ from traditional catching-type gauges (CGs) because they measure the diameter of each hydrometeor individually when it crosses the sensing volume of the instrument with no need to collect rainwater in a container. By using indirect methods based on laser beams, optical imaging, impact transducers, and acoustic or radar sensors, they often allow the joint detection of the hydrometeor size and fall velocity.

According to the measuring principle exploited, NCGs have a complex, often non-axisymmetric outer design because of the geometric constraints imposed by the sensor used. The absence of a collector implies that the measuring volume is not always physically delimited, like for example in radar or optical scatter sensors. An important limitation is that NCGs, regardless of their measuring principle, generally assume that hydrometeors fall vertically at their terminal velocity for the purpose of signal pre-processing. This hypothesis is only accurate in controlled conditions or in the absence of wind, although in general it is not verified.

NCGs have several advantages over traditional CGs, for example they also provide, further to the precipitation rate, other relevant parameters like the particle size distribution or visibility at a high temporal resolution. Furthermore, the lack of moving parts and the reduced maintenance required make them especially suitable for use in harsh environments and automatic weather stations, fostering their incremental adoption. The most relevant factors still preventing a widespread diffusion of NCGs are the lack of standardised calibration procedures and correction algorithms to compensate for instrumental and environmental biases [1].

Among the environmental sources of bias, wind is the most relevant one [2], causing the so-called exposure effect [3]. The gauge body, immersed in a wind field, behaves like a bluff-body obstacle to the undisturbed airflow, producing strong velocity gradients, vertical components, and the development of turbulence close to the gauge surface, see, e.g., [4,5]. The hydrometeor trajectories are diverted by the velocity field around the instrument [6] depending on their diameter, the gauge shape, wind speed, and wind direction. The induced change in the number of hydrometeors that cross the sensing volume/collecting area of the gauge (for NCGs and CGs, respectively) can lead, in windy conditions, to an over or under estimation of the precipitation amount and intensity (see, e.g., [7]).

The exposure effect therefore introduces a measurement bias, common to all precipitation gauges, simply because of the presence of the instrument itself (invasive measurement). This effect, well documented in the literature for CGs, is amplified in the case of NCGs due to their complex shape and measurement principle. The non-axisymmetric shape of the gauge implies a dependency of the aerodynamic effect on the wind direction. To reduce the wind induced bias, hardware solutions involve shielding the gauge against the incoming airflow, e.g., by inserting it in a pit or using a wind shield [8,9]. Although effective, this approach is usually viable only in test sites, for research applications, while it is less practical for operational meteorological stations, especially in urban areas where the available space is often limited.

To quantify the airflow deformation near the gauge, field campaigns, wind tunnel (WT) testing and numerical simulation can be employed. Experimental approaches are expensive in terms of time and resources and are strictly limited to the experienced precipitation and wind climatology at the experimental site. As an alternative, numerical simulation allows us to investigate various configurations in terms of wind velocity and direction, gauge shape, and precipitation type, see, e.g., [5,10], within limited costs and time. These are based on computational fluid dynamic (CFD) models that investigate the airflow velocity pattern induced by the instrument.

In the case of CGs, a wide literature on the numerical approach is available. This was adopted for the first time by Nešpor and Sevruk [11] on gauges with cylindrical shape, characterized by various design and thickness of the collector. CFD finite-volume simulations were run, solving the three-dimensional Reynolds-averaged Navier–Stokes (RANS) equations by means of the k-ε turbulence closure model (where k is the turbulent kinetic energy and ε is the turbulent dissipation per unit mass). The limited computational resources available at that time forced the adoption of a coarse computational mesh. Thériault et al. [12] performed RANS k-ε simulations on a shielded weighing gauge with chimney shape. In the work of Colli et al. [13,14,15], after increasing the detail of the computational mesh, both RANS simulations with a k-ω shear stress transport (SST) closure model [16] (with ω being the turbulent specific dissipation rate) and large eddy simulations (LES) were performed on the same gauge geometry.

To the authors’ knowledge, the only previous attempt to investigate the wind-induced bias on NCGs was presented by Nešpor et al. [17]. In that work, the basic method and the computational capabilities were the same as Nešpor and Sevruk [11]. Quite a coarse computational mesh was implemented, while no validation of the CFD results against WT measurements was provided. A two-dimensional video disdrometer (2DVD) was studied, but its simulated outer shape is now obsolete and differs significantly from the current version of the instrument. The 2DVD is a very special case of NCG, mainly used in field test sites for research purposes, while different NCGs are generally used at operational measurement sites. The geometry of the 2DVD is also rather simplified with respect to typical NCGs and the work of Nešpor et al. [17] cannot be extrapolated to provide information about the complex outer shape of other optical disdrometers.

An example of the observed wind-induced effects for a specific NCG is shown in the work of Friedrich et al. [18], where particle size velocity laser disdrometers (PARSIVEL, manufactured by OTT Hydromet) in a typical stationary installation were compared with others having an automatic variable orientation and tilting capacity, according to the wind direction. Under high-speed wind and heavy rain conditions, the authors reported artifacts in the measured drop size distribution from the stationary disdrometers that were not observed when the instrument sampling area was rotated into the wind.

In this work, we performed numerical simulation of the wind-induced airflow pattern near the sensing area of the laser precipitation monitor (LPM), manufactured by Thies Inc. This NCG is widely used by researchers, see, e.g., [19,20,21,22], and is being progressively integrated in NWS precipitation measurement networks, thanks to its performance and relatively low cost [23].

Field comparisons, like the work of Upton and Brawn [24], show that, even at limited wind speed, two such instruments installed with two different orientations (rotated by 90°) may report differences of up to 20% in the total number of detected hydrometeors This suggests that the wind direction, further than the wind speed, also affects their operational performance.

## 2. Methodology

**Figure 1:**
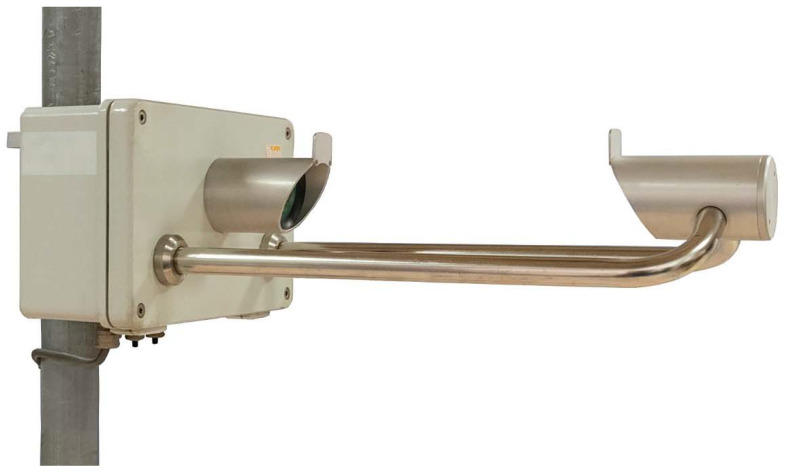
The Thies LPM [25], with the emitting head (attached to the circuitry box on the left-hand side) and the receiving head (attached to the supporting arms on the right-hand side).

The Thies LPM employs a laser beam to detect hydrometeors in flight. As shown in Figure 1, the instrument body is composed of a prismatic housing for the circuitry boards, with attached light emitting head and two supporting arms. Located at the end of the two arms, and aligned with the emitting head, is the receiving sensor. The instrument uses an infrared (785 nm) laser diode, coupled with suitable optics, to produce an infrared light sheet 228 mm long and 20 mm wide with a thickness of 0.75 mm. The laser emission is maintained at a constant frequency of 173 kHz and a low-pass filter is used to minimize interference from the ambient light. A photodiode in the receiving head is then used to convert the laser beam power into an electric signal.

When each single hydrometeor crosses the beam, the receiver will detect a reduction in the laser power and therefore a reduction in the electric signal. The diameter of the detected particle is calculated as a function of the voltage drop, while its fall velocity is obtained from the duration of the voltage reduction. The combined values of the diameter and fall velocity at one-minute resolution are used to define the precipitation type (drizzle, rain, snow, soft hail, hail, and mixed precipitation), and to derive the precipitation intensity. Velocity is also used to automatically discard false readings produced by objects like insects or falling leaves that may cross the beam. The system cannot differentiate between multiple hydrometeors simultaneously crossing the laser beam; therefore, these may be interpreted as a single particle of a larger diameter or be discarded by the instrument software, leading to an overestimation or underestimation of the total water volume. Another similar, but opposite, error is due to hydrometeors crossing the beam near its edges, blocking the laser beam only partially, which are detected as smaller particles leading to some underestimation of the water volume.

Note that installation instructions from the manufacturer [25] indicate that the orientation of the instrument in the field should be with the main symmetry axis aligned with the North direction, and the receiving end toward the equator (to minimize the effect of direct sunlight on the sensor), meaning that it is not possible to orientate the instrument according to the prevalent wind direction at the installation site.

In this work, CFD simulations were performed to investigate the airflow pattern near the Thies LPM outer geometry, using the open source OpenFOAM software package. Various undisturbed wind velocity values were tested while, due to the non-axisymmetric shape of the instrument body, multiple simulations were run by varying the incoming wind direction [18]. Additionally, the supporting pole of the instrument was included in the computational domain, since for some wind directions it may have an influence on the airflow velocity magnitude and direction at the sensing area.

The unsteady Reynolds-averaged Navier–Stokes (URANS) equations were numerically solved. Pseudo-transient simulations based on a local time stepping (LTS) numerical scheme [26] were performed to reduce the computational burden. In this approach the simulation is forced toward a steady state condition similarly to the Reynolds-averaged Navier–Stokes (RANS) approach, but with no need to remove the time derivative in the Navier–Stokes equations, therefore improving stability especially for complex geometries. This is obtained by setting a different time step for each cell of the computational domain, according to the imposed value of the Courant number, therefore enhancing numerical convergence.

Simulations were then validated by using wind tunnel (WT) measurements to verify that the complex airflow pattern generated by the instrument is correctly reproduced.

### 2.1. Fluid Dynamics Simulation Setup

A numerical model of the Thies LPM, including the supporting pole, was realised in the Standard Triangulation Language (STL) format. The computational mesh was produced within OpenFOAM, for a 4 m long, 2.4 m wide, and 2 m high simulation domain. The longitudinal axis (X) is set along the main symmetry axis of the instrument, the vertical axis (Z) is directed upward, while the Y axis is normal to the (X, Z) plane. The origin of the reference system is in the centre of the sensing area (the infrared light sheet). The internal mesh has a maximum cell size of 0.04 m and is progressively refined (up to 1 mm near the instrument walls) to reproduce the finer geometrical details and to correctly model the turbulence generated by the gauge-flow interaction.

The direction of the incoming wind was initially set parallel to the X axis, while nine different meshes were realized to simulate various wind directions, from α = 0° to α = 180° with increments of 22.5°, where α is the angle between the wind direction and the main symmetry axis of the instrument. In the configuration at α = 0°, the wind impacts first on the receiving sensor while, in the configuration at α = 180°, it first impacts on the supporting pole and the circuitry box. For each wind direction, five wind speed values (U_ref_) equal to 2, 5, 10, 15, and 20 m/s were tested for a total of 45 simulated wind direction/velocity configurations.

To evaluate the mesh resolution requirements, preliminary simulations conducted for a limited run time were used to calculate the ratio between the integral length scale and the grid length scale, named R*_L_*. This parameter represents, for each cell, the ratio between the turbulence length scale and the cell size. It is a common CFD practice to refine the mesh in the zones of interest using R*_L_* as a reference. For URANS simulations, it is advisable to keep R*_L_* ≥ 5, so that the larger eddies, adding-up to 80% of the turbulence kinetic energy, are discretized by at least 5 cells [27].

In the present work, the k-ω shear stress transport (SST) turbulence model is used, where k is the turbulent kinetic energy and ω the specific turbulent dissipation rate. Therefore, the integral length scale (*L_0_*) is calculated from Equation (1) [28], while R*_L_* is calculated from Equation (2).

(1) L0=k12Cμ·ω

(2) $$R_{L}=\frac{L_{0}}{\sqrt[{3}]{V}}\;$$

where the coefficient *C_µ_* is equal to 0.09 and *V* is the volume of the cell.

**Figure 2:**
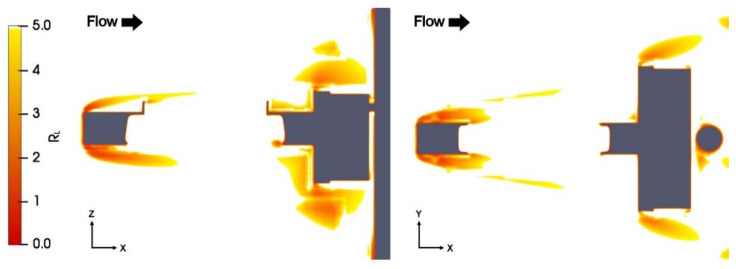
Maps of the R_L_ ratio at U_ref_ = 10 m/s for α = 0°, along the (X, Z) section at Y = 0 (left-hand panel) and (X, Y) section at Z = 0 (right-hand panel).

The computational mesh was progressively refined until the criterion R*_L_* ≥ 5 was satisfied in most of the domain. As shown in the maps of Figure 2, along the (X, Z) and (X, Y) sections of the domain at Y = 0 and Z = 0, respectively, only in the proximity of the straight and sharp edges the R*_L_* criterion could not be met.

For the nine wind directions investigated, the final mesh contains between four and five million cells and the values of typical mesh size and quality parameters, including the non-orthogonality, skewness, and aspect ratio, are listed in Table 1. These are used to identify highly distorted cells that could affect the solution and their value should be as low as possible. In Table 1, the parameters for all meshes are shown to fall inside the range that is considered acceptable for external aerodynamic simulations. A more in-depth explanation can be found in Aqilah et al. [29] and Baker [30].

**Figure 3:**
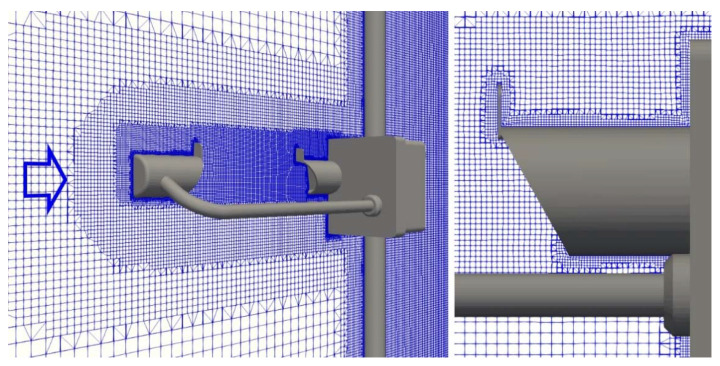
The computational mesh along the longitudinal cross-section of the domain around the Thies LPM at Y = 0 for the configuration at α = 0° (left-hand panel). The arrow indicates the direction of the incoming undisturbed wind flow. Close-up details of the mesh near the instrument emitting head (right-hand panel).

A sample (X, Z) section of the mesh at Y = 0 is presented in Figure 3 for the configuration at α = 0°, with details of the region close to the surface of the instrument emitting head. Coarser meshes were also tested, using a slightly simplified version of the geometry that allowed us to reduce the cell count to about 1M elements, while still preserving the instrument features. These meshes, however, failed to meet the R_L_ criterion and showed poor agreement with the WT measurements. It can be concluded that, for the geometry used in this work, no meaningful reduction of the cell count can be achieved without sacrificing the simulation accuracy.

Simulations were conducted by setting air as an incompressible fluid, with a density of 1.0 kg/m^3^ and a kinematic viscosity of 1.5 × 10^−5^ m^2^/s. The free stream turbulence intensity was set equal to 1%. From preliminary simulations the dimensionless wall distance (y+) was evaluated. Depending on the wind velocity, its average value indicates whether the cells closest to the instrument surface are positioned in the buffer or the log-law layer. Appropriate wall functions (independent on y+) were used for k, ω, and the turbulent viscosity (ν_t_) as near-wall boundary conditions at all solid surfaces [31].

### 2.2. Wind Tunnel Tests

**Figure 4:**
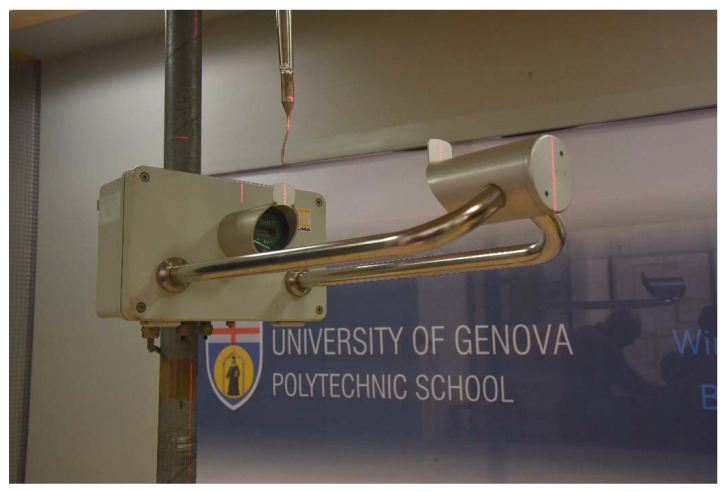
The Thies LPM during the installation procedure in the DICCA WT for the configuration at α = 0°. The instrument and the Cobra probe are aligned by employing a laser beam.

The experimental campaign was conducted in the WT facility available at the Department of Civil, Chemical and Environmental Engineering (DICCA) of the University of Genova. Measurements were taken using a multi-hole pressure probe, called “Cobra”, attached to a traversing arm with three-degrees of freedom. By measuring the local pressure, the Cobra probes provide the three velocity components of the flow, in a range between 2 and 100 m/s. [32]. A full-scale Thies LPM instrument was installed on its supporting pole in the WT, fixed to a rotating baseplate. A laser beam was used to check the alignment between the longitudinal axis of the instrument and the head of the Cobra probe, fixed to the traversing system (see Figure 4). This operation was repeated by rotating the instrument around the supporting pole, for each of the nine wind directions investigated, to ensure that the WT flow did impact the instrument at the exact angle chosen for the numerical simulation.

For each rotation, the airflow velocity was set equal to 5 and 10 m/s and for α = 0°, 45°, and 90° a reduced number of probe positions were sampled also at U_ref_ equal to 3, 7.5, and 15 m/s, to investigate the scalability (Reynolds dependency) of the flow field.

**Figure 5:**
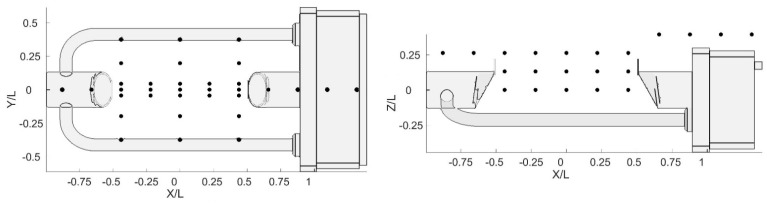
Normalized positions (black dots) of the Cobra probe measurements during the WT tests.

For all wind directions, the flow velocity was measured at the positions indicated with black circles in Figure 5, where the normalized sections (X/L, Z/L) and (X/L, Y/L) are depicted, and L = 0.228 m is the length of the sensing area of the instrument. For each position, measurements were taken at a frequency of 1000 Hz for 30 s. In total, 915 flow velocity measurements were obtained for nine rotations and two undisturbed airflow velocities.

## 3. Results

In this section, CFD simulation results are shown in terms of maps of the normalized magnitude and vertical component of the flow velocity (indicated with U_mag_/U_ref_ and U_z_/U_ref_, respectively) and maps of the normalized turbulent kinetic energy (k/U_ref_^2^). A 3-D visualisation of the turbulent structures, using the Q-criterion, is also included. CFD velocity profiles are compared with WT measurements for the whole set of investigated wind velocity and directions, and results are summarized in the form of basic statistics for selected performance parameters.

### 3.1. CFD Simulation Results

**Figure 6:**
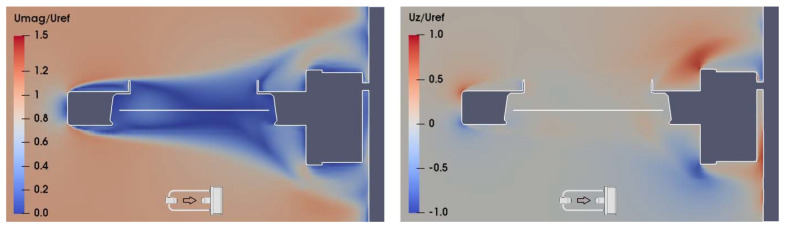
CFD simulation at U_ref_ = 10 m/s and α = 0°; maps of the normalized magnitude U_mag_/U_ref_ and vertical component U_z_/U_ref_ of the flow velocity (left- and right-hand panel, respectively), along the (X, Z) section of the flow field at Y = 0. The white horizontal line indicates the position of the sensing area of the instrument, while the small arrow indicates the undisturbed flow direction.

**Figure 7:**
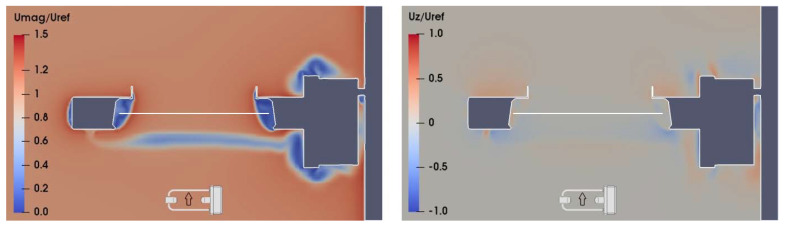
CFD simulation at U_ref_ = 10 m/s and α = 90°; maps of the normalized magnitude U_mag_/U_ref_ and vertical component U_z_/U_ref_ of the flow velocity (left- and right-hand panel, respectively), along the (X, Z) section of the flow field at Y = 0. The white horizontal line indicates the position of the sensing area of the instrument, while the small arrow indicates the undisturbed flow direction.

**Figure 8:**
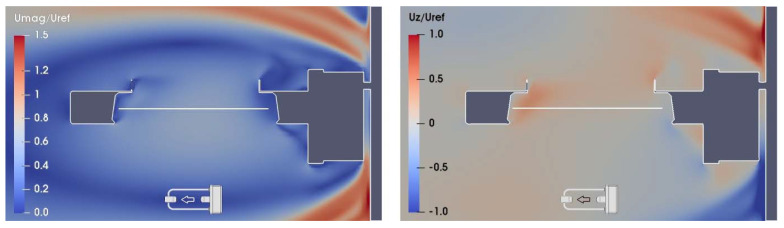
CFD simulation at U_ref_ = 10 m/s and α =180°; maps of the normalized magnitude U_mag_/U_ref_ and vertical component U_z_/U_ref_ of the flow velocity (left- and right-hand panel, respectively), along the (X, Z) section of the flow field at Y = 0. The white horizontal line indicates the position of the sensing area of the instrument, while the small arrow indicates the undisturbed flow direction.

As a sample of the large numerical dataset obtained from CFD simulations, wind velocity maps in the (X, Z) section of the flow field at Y = 0, for U_ref_ = 10 m/s and α = 0°, 90°, and 180° are shown in Figure 6, Figure 7 and Figure 8, respectively. In the left-hand panels, the red zones indicate a larger flow velocity than the undisturbed wind speed, therefore U_mag_/U_ref_ > 1, while in the blue zones the flow velocity is lower than the undisturbed wind, and U_mag_/U_ref_ < 1. In the right-hand panels, the red zones indicate upward flow velocity components, with U_z_/U_ref_ > 0, while downward components occur in the blue zones, where U_z_/U_ref_ < 0.

**Figure 9:**
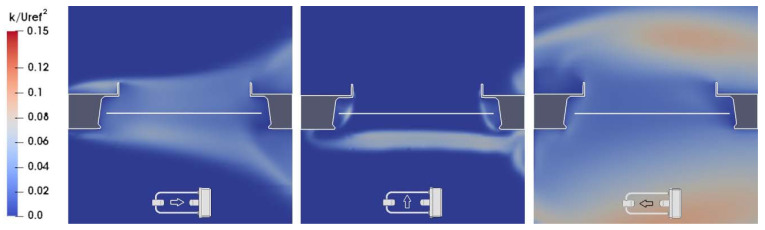
Maps of the normalized turbulent kinetic energy (k/U_ref_^2^) from CFD simulations at U_ref_ = 10 m/s along the (X, Z) section of the flow field at Y = 0 and for α = 0°, 90°, and 180° in the left-, central, and right-hand panels, respectively. The white horizontal line indicates the position of the sensing area of the instrument, while the small arrow indicates the undisturbed flow direction.

In Figure 6, at α = 0°, the receiver head is the first bluff-body obstacle to the flow, which generates accelerated zones (left-hand panel) and vertical velocity components (right-hand panel) above and below the sensing area of the instrument (white horizontal line), and a recirculation zone just downstream of the obstacle, with reduced velocity and high turbulence (as shown in the left-hand panel of Figure 9).

In Figure 7, at α = 90°, the flow near the sensing area is mostly undisturbed. The shedding of vortices generated by the supporting arms produces only a limited influence on the velocity magnitude and remains below the sensing area, while recirculation zones are concentrated near the receiving head, the instrument box, and the emitting head. Vertical velocity components are present only close to the instrument body, except for a limited amount of downdraft and updraft, generated by the vortex shedding, below the sensing area. Turbulence close to the laser beam is minimal (see the central panel of Figure 9).

In Figure 8, at α = 180°, the circuitry box acts as a large bluff-body obstacle for the flow and, together with the supporting pole, generates a large recirculation zone completely enclosing the instrument body. Above the sensing area, two zones of first accelerated and then decelerated flow are present, with a considerable updraft and generation of turbulence due to the recirculation effect (see the right-hand panel of Figure 9).

**Figure 10:**
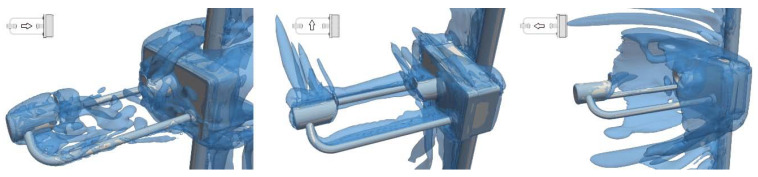
Visualization of the turbulent structures around the instrument using the Q-criterion at U_ref_ = 10 m/s and α = 0°, 90°, and 180° in the left-, central, and right-hand panels, respectively.

The presence of turbulence and vortex structures near the instrument body is visualized in Figure 10 by means of the Q-criterion [33]. At α = 0° (left-hand panel), the wake generated by the instrument receiving head is highlighted, affecting the sensing area of the instrument and the flow region above it. At α = 90° (central panel), neither the turbulence structures produced by the two heads and the circuity box, nor the turbulent wake produced by the supporting arm affect the sensing area of the instrument. At α = 180° (right-hand panel), the circuitry box produces large vortex structures that completely envelop the instrument sensing area.

Similar results were obtained at further wind velocity and directions. Recirculation and vertical velocity components near the sensing area non-linearly decrease from the 0° configuration to the 90° configuration, where a minimum is reached, and then increase again approaching the 180° configuration, where the maximum amount of flow disturbance is obtained.

### 3.2. Analysis of WT Measurements

The Cobra pressure probes used in the WT allow us to perform some quality checks on the measured data. First, the probe returns a null value in case of relevant airflow components reaching the probe from outside of a 45° acceptance cone, since the positioning of the pressure holes on the probe head does not allow detection of the flow velocity in such conditions. This is typical of recirculating flow and strong turbulence conditions. Second, the probe has a lower sensitivity of about 2 m/s, below which the measurement is deemed unreliable.

For each position sampled in the WT experiment, an assessment of the measurement quality was therefore performed by counting the number of null values obtained during the 30 s acquisition time frame. The measurement was discarded in case null values exceeded 20% of the total sample. Measurements were also discarded when falling below the minimum velocity of 2 m/s or presenting a turbulent intensity larger than 30%, as indicated by the probe manufacturer specifications.

The measured turbulence intensity and percentage of discarded measurements are shown in Table 2. As expected, the most critical configuration is at α = 180°, where the combined effect of the circuitry box and the supporting pole produces strong recirculation zones and high turbulence intensity, with almost all measurements being rejected. On the other hand, the 67.5°, 90°, and 112.5° configurations, because of their favourable cross section obstacle presented to the incoming flow, are the least disturbed, with all measurements satisfying the quality, velocity, and turbulence intensity criteria.

### 3.3. Validation

**Figure 11:**
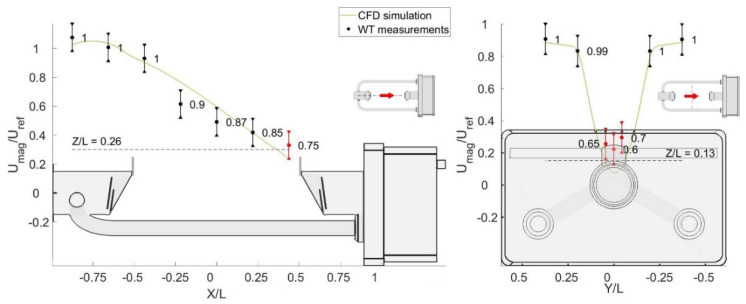
Comparison of the simulated profiles of the magnitude of flow velocity against WT measurements for the configuration at α = 0° and U_ref_ = 5 m/s.

**Figure 12:**
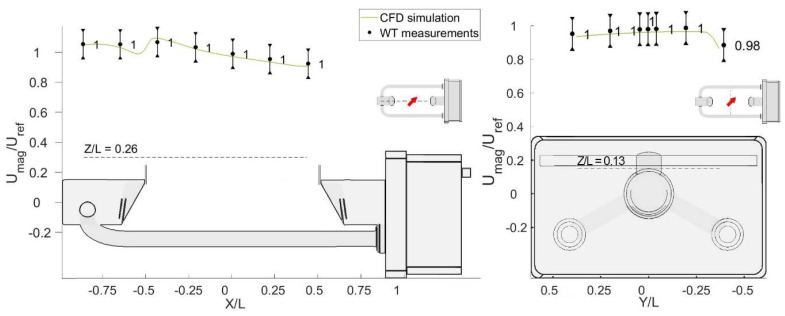
Comparison of the simulated profiles of the magnitude of flow velocity against WT measurements for the configuration at α = 45° and U_ref_ = 5 m/s.

**Figure 13:**
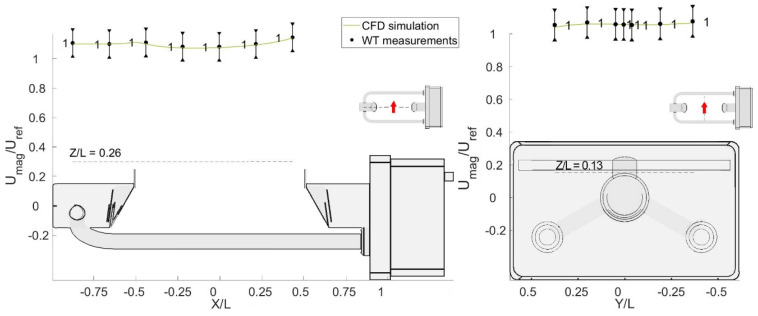
Comparison of the simulated profiles of the magnitude of flow velocity against WT measurements for the configuration at α = 90° and U_ref_ = 5 m/s.

Validation of the simulation results was performed by comparing the average simulated velocity with the measurements obtained in the WT experiment. In Figure 11, Figure 12 and Figure 13, simulated profiles and measured data are reported. Error bars represent the measurement tolerance specified by the probe manufacturer, while for each WT probe a quality index is shown both numerically and by colour coding. This index is defined as the ratio between the number of samples correctly acquired and the total number of samples. Therefore, unity indicates valid measurements where no null values are present, while zero indicates that all values are null. The simulation is shown to be in very good agreement with the WT measurements for the three angles reported.

Due to the large number of measurements taken, a statistical approach was also used, whose results are presented in Table 3. For this analysis, only the 661 measurements satisfying the quality (>0.8), velocity (>2 m/s), and turbulence intensity (<0.3) criteria are considered. The percentage of samples that fall within 2σ and 3σ was calculated, with σ being the instrument accuracy (0.5 m/s). Under the hypothesis of a Gaussian distribution of measurement errors, 95.45% of the points are expected to be within 2σ, while 99.73% should be within 3σ.

Most rotations satisfy this criterion, although with a few outliers. These could be explained by the fact that some points still present a high turbulence intensity that is barely below the imposed limit and that the presence of the probe itself locally modifies the velocity field, especially near the surface of the instrument. Additionally, because of the limited number of usable data for some angles, one single outlier could have a strong influence on the test result.

The influence of a limited number of outliers is reduced when considering the whole dataset. Only 44 out of the 661 measurements considered valid, 6.65% of the total, present a difference with respect to the simulation results larger than the instrument tolerance of 0.5 m/s. The numerical simulation model is therefore capable of correctly capturing the phenomenon, especially for the more favourable configurations where the agreement is almost perfect, providing satisfactory results even in less favourable conditions of high turbulence and recirculating flow.

### 3.4. Reynolds Number Dependency

**Figure 14:**
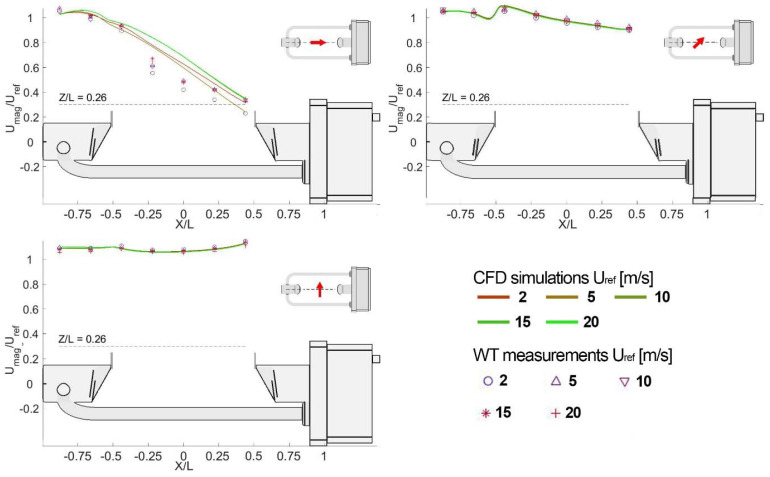
Reynolds number dependency of four simulated profiles (at U_ref_ = 2, 5, 10, 15, and 20 m/s) of the normalised magnitude of the airflow velocity at Z/L = 0.26 (dashed line) above the measuring area and WT measurements along the same profile at 3, 5, 7.5, and 15 m/s.

A scalability analysis was conducted to assess the validity of the solution at airflow velocities in between the simulated ones. Three further velocities (3, 7.5 and 15 m/s) were investigated to this end in the WT for three different wind directions (0°, 45° and 90°) and a few simulated wind velocity profiles, at Z/L = 0.26 above the instrument measuring area, are illustrated in Figure 14. For α = 45° and α = 90°, both the simulated profiles and measured data show almost no Reynolds number dependency, while for the 0° configuration some differences occur in both the measured data and simulated profiles in the areas with a higher turbulence intensity.

### 3.5. Analysis of the Flow Field Near the Sensing Area

To compare the effects of the wind between different instrument configurations, a control volume is defined—just above the sensing area—as a box with a length of 0.228 m, a height of 0.1 m, and a width of 0.05 m. The lower face of the control volume coincides with the laser beam. This volume represents the portion of the wind field having a strong potential to influence hydrometeors when approaching the instrument sensing area and provides an overall indication of the wind field deformation due to the presence of the instrument body.

A summary of the normalised velocity components and turbulent kinetic energy obtained from the CFD simulation within the control volume is presented in this paragraph. The average of the results obtained for the five wind speed values simulated is reported for each wind direction. Additionally, the percentage of the control volume where updraft or downdraft is observed is shown, defined as the ratio between the volume of the cells where vertical velocity is positive (updraft) or negative (downdraft) and the total size of the control volume.

Maximum and average values of the vertical velocity components are reported in Table 4. It is confirmed that the lower impact is associated with the two configurations at 0° and 90° (and nearby angles). Maximum values are in general higher when the wind impacts at an angle with respect to the instrument axis. The 22.5°, 45°, 135°, and 157.5° configurations produce the highest values, because the flow is not blocked like in the 0° or 180° configurations, differently from angles close to 90° where the instrument presents the most favourable cross-section obstacle to the flow.

In terms of average values and extension (percentage within the control volume), updraft is predominant (see the second-last column). The only two exceptions are the 135° and 157.5° configurations, where the circuitry box, with its large, slanted, bluff-body obstruction to the flow, produces predominant downdraft components in the control volume (see last column).

Table 5 shows the horizontal velocity components. As expected, the longitudinal velocity is predominantly influenced by the flow blockage due to the instrument body. Maximum and average longitudinal velocities gradually increase from the 0° configuration to the 90° configuration, then decrease towards the configuration at 180°. A sudden jump is evident between 157.5° and 180° due to the impact of the circuitry box that produces very strong recirculation, which is also responsible of the change of sign of the average velocity. The minimum longitudinal velocity shows quite limited variations, with comparable backward components between the various configurations, except at α = 180°.

Transversal velocity components have a clearly different behaviour. For the 0° and 180° configurations, the average velocity is close to zero to indicate, as expected, that the flow is symmetrical, and the maximum and minimum values are limited. The other configurations present higher values of maximum, minimum, and average transversal velocity. At α = 90°, where the gauge surfaces are mostly parallel or perpendicular to the flow, high maximum and minimum but low average values are obtained, because transversal velocity components are only present near the instrument receiving and transmitting heads.

Table 6 shows the standard deviation of the normalised velocity components and turbulent kinetic energy. The standard deviation of the longitudinal component suggests that the flow is more uniform for angles near 90° and that the strong recirculation observed at α =180° is relatively uniform close to the instrument sensing area. The standard deviation for both the transversal and vertical velocity components shows that the least uniform fields occur for shallow angles between the wind direction and the main symmetry axis of the instrument.

The maximum turbulent kinetic energy has a limited variability, while the average values change significantly depending on the wind direction. This means that the maximum energy of the generated eddies does not change much with the wind direction, but the number and distribution of those eddies (and therefore the overall turbulence) is strongly affected by the instrument blockage. The standard deviation of the turbulent kinetic energy shows the highest values at α = 22.5° and 135°.

## 4. Discussion

Both simulation results and WT measurements show that wind direction is the primary factor dictating the airflow pattern near the Thies LPM, even more so than the wind speed, due to the demonstrably limited Reynolds number dependency. The airflow near the sensing area changes considerably and, in some cases, abruptly with the wind direction, showing a strongly non-linear behaviour that makes it difficult to predict the effect of wind without dedicated CFD simulations.

Depending on the wind direction, near and above the sensing area the normalised average updraft is between 3% and 10% (with peak velocities up to 70% of the freestream value), while the normalised downdraft is between 2% and 16% (with peaks up to 40% of the freestream value). The horizontal velocity component also increases significantly, up to 27%, and even reverses its direction (with values up to 55% of the freestream velocity) due to the induced recirculation. Strong transversal velocity components are also present, with normalised peak values up to 88% and average values up to 10%. These strong velocity gradients near the instrument body are non-negligible and potentially affect the approaching hydrometeors. These are indeed slowed down by strong updraft components or diverted away from the sensing area by strong transversal velocity components. Turbulence also changes considerably depending on the wind direction, with configurations that on average reach up to 15 times the kinetic energy of the less impacting ones.

The most favourable configuration is at α = 90°, presenting the lowest value of turbulence and among the lowest values of both updraft and downdraft components. It is therefore expected that this configuration would introduce the least amount of bias in measurements taken under the influence of wind. Angles close to 90° also present favourable results while the configurations at 0° and especially 180° are the worst performing ones, with the latter producing the strongest impact on the nearby airflow conditions. The occurrence of such configurations should be minimised in field installations, and extreme care should be taken in analysing measurements taken in such conditions, since the associated wind-induced bias is expected to be significant.

## 5. Conclusions

The aerodynamic behaviour of the complex outer geometry of NCGs induces vertical and accelerated/decelerated velocity components above and within the sensing area of the instrument. The present work demonstrated that such components are significant in the case of the Thies LPM precipitation gauge and quantified their magnitude using CFD simulation, suitably validated against dedicated WT flow velocity measurements.

The wind direction is found to be the most relevant influencing factor in determining the magnitude of the airflow perturbation, due to the non-axisymmetric geometry of the gauge. This must be considered when interpreting measurements obtained in windy conditions, since the positioning of the instrument in the field is constrained by the sensor specifications and cannot be aligned with the predominant wind at the installation site to minimize this effect.

The observed airflow pattern generated by the gauge body is indeed expected to induce non-negligible bias in operational measurements, especially in strong wind and light precipitation. The proposed airflow numerical simulation framework provides a basis to develop correction curves for the wind-induced bias of NCGs, depending not only on the undisturbed wind speed and precipitation intensity, but also on the wind direction.

## Figures and Tables

**Table 1:** Mesh size and quality parameters for the nine configurations investigated.

Wind Direction	n° Cells	Avg. Non-Orthogonality	Max Non-Orthogonality	Max Skewness	Max Aspect Ratio
**0.0°**	4′022′992	6.44	54.87	3.45	13.35
**22.5°**	4′070′482	6.88	54.98	3.93	11.05
**45.0°**	4′144′606	6.81	55.46	3.96	13.28
**67.5°**	4′372′642	6.69	54.98	4.00	11.62
**90.0°**	4’626′698	6.19	54.98	3.45	13.35
**112.5°**	4’807′780	6.52	55.47	3.95	11.05
**135.0°**	4’943′269	6.45	55.47	3.95	13.28
**157.5°**	5’062′277	6.40	54.98	3.99	11.15
**180.0°**	5’081′405	5.99	54.88	3.45	13.35

**Table 2:** Quality parameters of the WT measurements.

**U_ref_ = 5 m/s**				
**Wind Direction**	**% Accepted**	**% Low Quality**	**%Low Speed**	**Mean Turb. Int.**
**0°**	36.92	49.23	60.00	0.376
**22.5°**	67.39	15.22	30.44	0.157
**45°**	95.75	4.26	4.26	0.048
**67.5°**	100	0	0	0.015
**90°**	100	0	0	0.017
**112.5°**	100	0	0	0.013
**135°**	82.50	10.00	17.50	0.094
**157.5°**	46.34	34.15	48.78	0.261
**180°**	0	82.35	94.12	0.646
**U_ref_ = 10 m/s**				
**Wind Direction**	**% Accepted**	**% Low Quality**	**%Low Speed**	**Mean Turb. Int.**
**0°**	43.08	44.62	46.62	0.353
**22.5°**	86.96	13.04	8.70	0.118
**45°**	95.75	4.26	2.13	0.037
**67.5°**	100	0	0	0.010
**90°**	100	0	0	0.015
**112.5°**	100	0	0	0.010
**135°**	87.50	12.50	10.00	0.095
**157.5°**	60.53	28.95	26.32	0.241
**180°**	20.00	60.00	80.00	0.422

**Table 3:** Statistics of the WT measurements.

**U_ref_ = 5 m/s**					
**Wind Direction**	**Mean Error [m/s]**	**Std. Dev. [m/s]**	**% Out of Tolerance**	**% Data within 2σ**	**% Data within 3σ**
**0°**	0.376	0.554	25.00	83.33	95.83
**22.5°**	0.345	0.538	12.00	92.00	92.00
**45°**	0.117	0.267	2.22	97.78	97.78
**67.5°**	0.103	0.192	4.08	100.00	100.00
**90°**	0.084	0.156	1.56	98.44	100.00
**112.5°**	0.102	0.245	2.22	97.78	97.78
**135°**	0.228	0.448	15.63	90.63	96.88
**157.5°**	0.322	0.446	10.53	94.74	100.00
**180°**	1.806	1.185	33.33	58.33	75.00
**U_ref_** **= 10 m/s**					
**Wind Direction**	**Mean Error [m/s]**	**Std. Dev. [m/s]**	**% Out of Tolerance**	**% Data within 2σ**	**% Data within 3σ**
**0°**	0.370	0.590	33.33	75.00	91.67
**22.5°**	0.410	0.351	15.79	92.11	97.37
**45°**	0.098	0.147	0.00	100.00	100.00
**67.5°**	0.157	0.280	4.44	97.78	100.00
**90°**	0.139	0.218	4.69	98.44	100.00
**112.5°**	0.121	0.151	0.00	100.00	100.00
**135°**	0.236	0.256	6.67	96.67	100.00
**157.5°**	0.540	0.758	27.78	83.33	88.89
**180°**	3.722	0.879	100	0.00	0.00

**Table 4:** Updraft and downdraft components within the control volume, obtained from CFD simulations as an average over the five wind speed values investigated.

Wind Direction	Max Updraft U_z_/U_ref_	Max Downdraft U_z_/U_ref_	Avg. Updraft U_z_/U_ref_	Avg. Downdraft U_z_/U_ref_	% Volume Updraft	% Volume Downdraft
**0°**	0.240	0.097	0.052	0.020	74.5	25.5
**22.5°**	0.629	0.386	0.095	0.052	79.3	20.7
**45°**	0.679	0.254	0.077	0.035	86.5	13.5
**67.5°**	0.410	0.150	0.057	0.022	91.3	8.7
**90°**	0.281	0.257	0.030	0.025	82.9	17.1
**112.5°**	0.553	0.158	0.041	0.030	92.9	7.1
**135°**	0.709	0.257	0.068	0.070	30.6	69.4
**157.5°**	0.649	0.410	0.068	0.159	3.2	96.8
**180°**	0.430	0.258	0.098	0.048	93.9	6.1

**Table 5:** Normalised longitudinal and transversal components within the control volume, obtained from CFD simulations as an average over the five wind speed values investigated.

Wind Direction	Max U_x_/U_ref_	Avg. U_x_/U_ref_	Min U_x_/U_ref_	Max U_y_/U_ref_	Avg. U_y_/U_ref_	Min U_y_/U_ref_
**0°**	0.995	0.328	−0.287	0.130	−0.001	−0.125
**22.5°**	1.036	0.629	−0.200	0.592	0.068	−0.472
**45°**	1.122	0.810	−0.299	0.643	0.097	−0.529
**67.5°**	1.163	0.956	−0.265	0.551	0.080	−0.647
**90°**	1.272	1.041	−0.287	0.656	0.025	−0.647
**112.5°**	1.236	0.936	−0.127	0.118	−0.048	−0.409
**135°**	1.203	0.663	−0.250	0.247	−0.099	−0.731
**157.5°**	1.092	0.366	−0.179	0.342	−0.097	−0.888
**180°**	0.172	−0.337	−0.551	0.255	0.001	−0.253

**Table 6:** Standard deviation of the normalised velocity components and statistics of the turbulent kinetic energy within the control volume, obtained from CFD simulations as an average over the five wind speed values investigated.

Wind Direction	Std. Dev. U_x_/U_ref_	Std. Dev. U_y_/U_ref_	Std. Dev. U_z_/U_ref_	Max k/U_ref_^2^	Avg. k/U_ref_^2^	Std. Dev. k/U_ref_^2^
**0°**	0.337	0.030	0.049	0.057	0.026	0.013
**22.5°**	0.345	0.115	0.110	0.084	0.012	0.020
**45°**	0.333	0.125	0.084	0.096	0.006	0.015
**67.5°**	0.257	0.111	0.058	0.086	0.003	0.009
**90°**	0.200	0.087	0.043	0.057	0.002	0.005
**112.5°**	0.292	0.064	0.047	0.067	0.007	0.014
**135°**	0.433	0.130	0.101	0.077	0.016	0.019
**157.5°**	0.346	0.199	0.111	0.057	0.030	0.015
**180°**	0.131	0.061	0.078	0.086	0.028	0.011

## Data Availability

Not applicable.
